# SPX-GNN: An Explainable Graph Neural Network for Harnessing Long-Range Dependencies in Tuberculosis Classifications in Chest X-Ray Images

**DOI:** 10.3390/diagnostics15243236

**Published:** 2025-12-18

**Authors:** Muhammed Ali Pala, Muhammet Burhan Navdar

**Affiliations:** 1 Department of Electrical and Electronics Engineering, Faculty of Technology, Sakarya University of Applied Sciences, Sakarya 54050, Türkiye; pala@subu.edu.tr; 2 Biomedical Technologies Application and Research Center (BIYOTAM), Sakarya University of Applied Sciences, Sakarya 54050, Türkiye; 3 Department of Civil Engineering, Faculty of Engineering, Sakarya University, Sakarya 54050, Türkiye

**Keywords:** graph neural networks, explainable AI, tuberculosis, deep learning, medical image

## Abstract

**Background/Objectives**: Traditional medical image analysis methods often suffer from locality bias, limiting their ability to model long-range contextual relationships between spatially distributed anatomical structures. To overcome this challenge, this study proposes SPX-GNN (Superpixel Explainable Graph Neural Network). This novel method reformulates image analysis as a structural graph learning problem, capturing both local anomalies and global topological patterns in a holistic manner. **Methods**: The proposed framework decomposes images into semantically coherent superpixel regions, converting them into graph nodes that preserve topological relationships. Each node is enriched with a comprehensive feature vector encoding complementary diagnostic clues, including colour (CIELAB), texture (LBP and Haralick), shape (Hu moments), and spatial location. A Graph Neural Network is then employed to learn the relational dependencies between these enriched nodes. The method was rigorously evaluated using 5-fold stratified cross-validation on a public dataset comprising 4200 chest X-ray images. **Results**: SPX-GNN demonstrated exceptional performance in tuberculosis classification, achieving a mean accuracy of 99.82%, an F1-score of 99.45%, and a ROC-AUC of 100.00%. Furthermore, an integrated Explainable Artificial Intelligence module addresses the black box problem by generating semantic importance maps, which illuminate the decision mechanism and enhance clinical reliability. **Conclusions**: SPX-GNN offers a novel approach that successfully combines high diagnostic accuracy with methodological transparency. By providing a robust and interpretable workflow, this study presents a promising solution for medical imaging tasks where structural information is critical, paving the way for more reliable clinical decision support systems.

## 1. Introduction

Tuberculosis, caused by the Mycobacterium, is one of the deadliest infectious diseases worldwide [1]. According to the World Health Organisation, millions of new cases are reported each year, and early and accurate diagnosis is critical to controlling the spread of the disease [2,3,4]. In this diagnostic process, especially in resource-limited regions, chest radiography is the most commonly used, rapid, cost-effective primary screening tool. However, the radiographic findings of tuberculosis can be quite varied and sometimes nonspecific, including consolidation, cavitation, pleural effusion, and miliary patterns. This situation makes interpreting radiological images a complex task that requires expertise and presents significant inter-observer variability.

To overcome these challenges in processing medical data and increase efficiency and consistency in diagnostic processes, machine learning-based algorithms have emerged as a promising solution [5,6,7,8]. Convolutional neural networks, which are deep learning architectures, enable the resolution of numerous problems in analysing radiological images [9,10]. Architectures such as convolutional neural networks eliminate complex feature engineering steps thanks to their ability to automatically learn hierarchical and diagnostically meaningful features directly from pixel data. Their ability to recognise low-level patterns, such as edges and textures, in early layers and to combine these patterns in deeper layers to identify more complex structures, such as nodules or cavities, has made them powerful tools in analysing medical data [11]. Deep learning architectures can be categorised based on how they perceive and process data. First, some architectures assume images are a regular and structured Euclidean grid formed by pixels and base their operations on local processing within this grid. On the other hand, some approaches move away from this grid assumption and aim to model the underlying, more flexible or relational data structures [12,13]. Architectures working on data in this grid structure are inherently insufficient in modelling long-range dependencies between distant regions of an image. To overcome this limitation, newer architectures such as the vision transformer offer a different approach by dividing the image into a series of parts and applying a self-attention mechanism between these parts [14].

Graph neural networks are deep learning architectures designed to process non-Euclidean data, specifically graph structures composed of nodes and the edges connecting them. Unlike CNNs and Recurrent Neural Networks, which traditionally work on structured data, graph neural networks base their fundamental operational principles on a principle that aggregates and transforms information from neighbouring nodes in a recursive way [15,16,17]. This structure enables graph neural networks to learn not only the attributes of the nodes themselves, but also the topological structure of the graph and the complex relational patterns between entities. Thanks to these capabilities, they offer robust solutions to problems where traditional deep learning methods fall short, spanning various applications from social network analysis to molecular chemistry and drug discovery, from knowledge graphs to recommendation systems and financial fraud detection [18,19]. Therefore, graph neural networks play an increasingly central role in deep learning as a fundamental tool for modelling complex interaction systems. GNNs begin by revealing the intrinsic structure of an image rather than imposing an external structure onto it. After the image is decomposed into semantic units using superpixels or cell segmentation methods, a graph is constructed based on the neighbourhood or interaction relationships between these units. Thus, the learning process takes place directly on a graph that reflects the natural topology of the data itself, rather than on an artificial grid or array that facilitates computation. This shifts the model’s inductive bias from the local arrangement of pixels to the morphology and organisation of biologically meaningful entities, thereby enabling the explicit modelling of complex structural information such as tissue architecture or the cellular microenvironment.

Although deep learning methods have achieved the most advanced performance in medical image analysis, their effectiveness is inherently limited by the local nature of convolutions or other operators. This architectural limitation restricts their ability to model long-range spatial dependencies and contextual relationships between distant regions in the data, often critical for a definitive diagnosis. These connections in the data must be meaningfully evaluated to tackle a crucial problem, such as tuberculosis diagnosis. In the case of tuberculosis, the diagnostic value of a slight opacity in the apex of the right lung can increase significantly when evaluated in conjunction with another finding in the lower lobe of the left lung. A standard deep learning approach is not explicitly designed to model such long-range spatial dependencies. This prevents the model from fully grasping the overall presentation of the disease, which is often multifocal and diffuse, and causes it to reach a plateau in diagnostic accuracy.

Our hypothesis for SPX-GNN is as follows: By transforming a medical image into a semantic graph where each node carries a rich feature signature of the underlying tissues, we can develop a superior diagnostic model to understand local abnormalities and learn global relationships in a deeper manner. This approach enables the model to handle uncertainty better and make robust diagnostic inferences. The fundamental and original contributions of this work to the literature are as follows:

1- Enriched Structural Graph Representation: A novel method is presented that converts medical images into a structural graph. Each superpixel node is transformed into a holistic feature vector encoding complementary diagnostic clues: colour and intensity variations in CIELAB space, texture pattern distortions using LBP and Haralick features, and shape anomalies using orientation-independent Hu moments. This deliberate fusion renders each node a richer, more interpretable entity than raw pixels.
2- Global Contextual Learning: SPX-GNN performs higher-level reasoning by learning relational patterns between these rich node representations. The model situates local findings within a broader context, examining the relationship between specific attribute anomalies and suspicious findings in other areas of the image.
3- Integrated Explainability: The framework includes an explainability module that generates node-level importance scores. This enhances clinical reliability by transforming the GNN’s decision-making process into intuitive importance maps, highlighting the specific anatomical regions contributing most significantly to the final diagnosis for concrete clinical decision support.
4- Demonstrated Practical Impact: The proposed method exhibits superior diagnostic performance with 98.7% accuracy, 96.1% F1-score, and a perfect 100.00% ROC-AUC, validating its effectiveness and reliability for real-world medical imaging tasks.

In this contribution, SPX-GNN presents a novel approach that simultaneously addresses the issues of locality and semantic gaps in medical image analysis by integrating multimodal feature extraction with global contextual learning, thereby enabling fully interpretable and holistic diagnostic inference.

## 2. Related Works

Deep learning models, particularly CNNs, provide significant accuracy in analysing radiographic images. The CNN–ViT model is a hybrid model that combines the complementary features of CNN and Vision Transformer architectures. By integrating ResNet-50 and ViT-b16 structures, the model accurately detects tuberculosis in chest X-rays and distinguishes between tuberculosis and pneumonia [20]. In a study comparing the VGG16, VGG19, ResNet50, ResNet101, ResNet152, and Inception-ResNet-V2 architectures, the performance of various CNN-based models was evaluated for detecting tuberculosis from chest X-ray images. The study reported that, based on experiments conducted on the dataset, the VGG16 model achieved the highest success rate and demonstrated performance similar to or superior to more complex models with lower computational costs [21]. AttCDCNet has been developed, based on the DenseNet121 architecture, with an added attention block to ensure the model focuses on the most meaningful regions. The focal loss function and deep separable convolutions have also been utilised to mitigate the imbalanced data distribution and reduce parameter load [22]. In another hybrid study, a hybrid artificial intelligence model combining the Vision Transformer and CNN architectures was developed as part of a study aimed at comprehensively analysing tuberculosis-related anomalies. The proposed model addresses a multi-class and multi-label classification problem, reducing data imbalance issues through data augmentation, class weighting, and focal loss methods [23]. A unified multi-task deep learning framework called CXR-MultiTaskNet has been developed; the model consists of a ResNet50-based feature extractor, two task-specific sub-heads for classification and localisation, and a Grad-CAM-based interpretability module to enhance explainability. Additionally, a combined loss function that emphasises representation and detection loss was used to reduce task imbalances within the model. The study achieved higher interpretability and accuracy than traditional CNN-based methods by producing explainable predictions at both the disease and pixel levels through dual-attention-based hierarchical feature extraction [24]. The diagnostic accuracy and interpretability have been enhanced by incorporating a Grad-CAM-based explainability module into the Vision Transformer architecture. The model can learn long-range dependencies and complex visual patterns directly from raw pixel information using a convolution-based preprocessing layer and multiple transformer encoder blocks. The Grad-CAM visualisations added to the developed model make the model’s decisions transparent, helping radiologists evaluate and validate AI-assisted diagnoses [14]. The proposed hybrid approach combines deep feature extraction with Vision Transformer, dimension reduction with PCA, and classification with various machine learning methods to enable fast and accurate detection of tuberculosis. The model was trained on the dataset after applying preprocessing steps of resizing, scaling, and noise reduction [25]. CoAtNet, a hybrid deep learning approach, combines the strengths of CNN and Vision Transformer architectures to enable the automatic classification of tuberculosis. The model is pre-trained on ImageNet to improve performance across different image types. Furthermore, LIME-based explainability integration makes model predictions transparent, delivering reliable and interpretable results [26]. In a hybrid approach, pre-trained CNN, transformer, and classical models were tested; the best individual performance was achieved with the CoAtNet model. Additionally, an ensemble approach was applied by combining the predictions of all models with weights determined by differential evolution [27]. A method called tbXpert, based on deep fused linear triangulation, has been developed. This method eliminates intra-class variations and inter-class similarities in images [28].

For deep learning-based diagnostic systems to establish trust in clinical applications, explainability alongside high accuracy has become a critical focus. It is imperative to be able to apply xAI methods in areas where diagnosis is quite challenging, such as tuberculosis. In this regard, techniques have been developed to classify various X-ray images and implement tuberculosis prediction using a CNN-based approach [29]. On the other hand, a DenseNet201-based transfer learning model has been developed for multi-lung disease classification, utilising the capabilities of DenseNet [30]. To ensure the interpretability of model decisions, XAI methods such as SHAP, LIME, Grad-CAM, and Grad-CAM++ were used. Another technique designed for tuberculosis detection based on U-Net has been proven to provide superior performance with segmented lung images compared to raw images [31]. The EfficientNetB3 model enabled the visualisation of tuberculosis-infected regions using visualisation metrics such as Grad-CAM on a combined dataset that integrated data augmentation and segmentation, thereby supporting diagnoses. Another method, a Shallow-CNN architecture, has been presented as more transparent in its interpretability compared to DenseNet, demonstrating its focus on lung regions critical for tuberculosis findings through evaluations using CAM and LIME techniques [32].

## 3. Proposed Method

**Figure 1:**
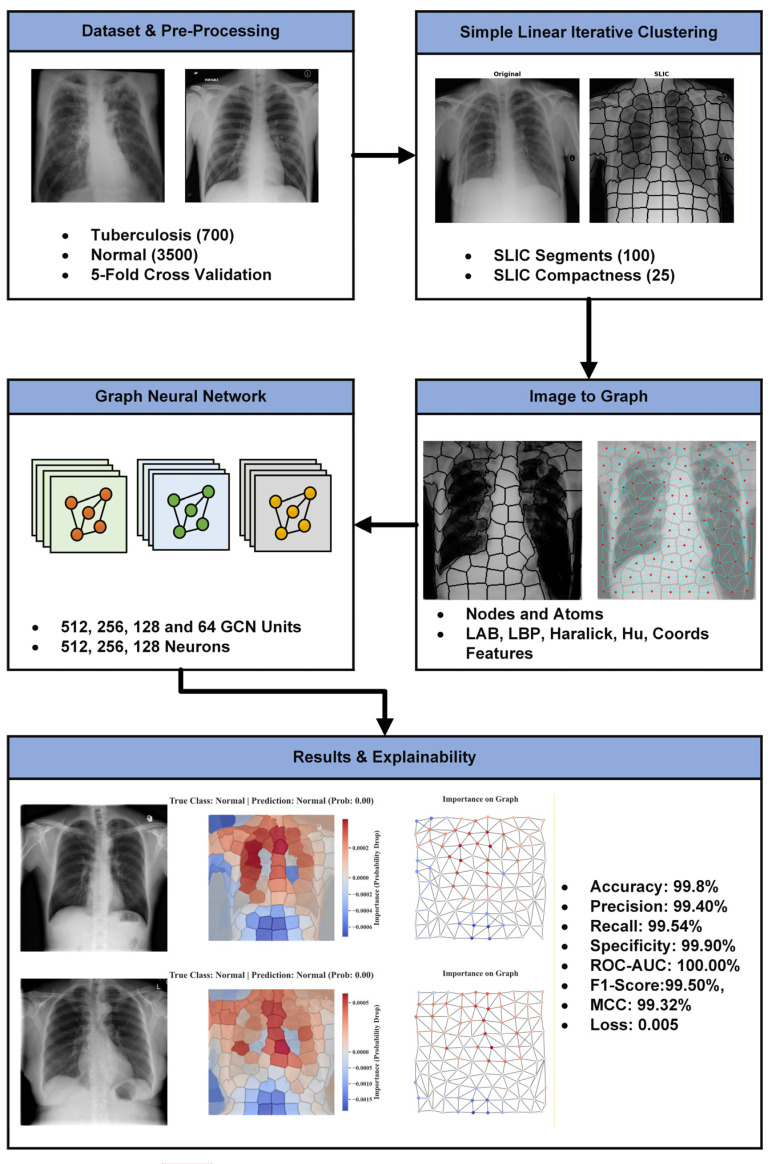
Flowchart of the SPX-GNN method.

This study proposes SPX-GNN, a super-pixel-based GNN designed to model long-range spatial dependencies and holistic context in medical images. The method aims to overcome the locality constraint of standard deep learning approaches by transforming the image from a Euclidean pixel grid to a semantic graph representation. SPX-GNN’s workflow consists of integrated core stages schematically shown in Figure 1: image-to-graph conversion, multi-directional node feature extraction, and graph-based classification.

The first step of the proposed method involves transforming unstructured pixel data into a structured and semantic form that the GNN can process. To this end, each input image is first decomposed into semantically coherent regions using the Simple Linear Iterative Clustering (SLIC) algorithm. SLIC produces perceptually meaningful and complementary superpixel areas based on colour/intensity and spatial proximity. Following this segmentation, a graph structure is created where the nodes represent the superpixels and the edges represent the spatial neighbourhood relationships between these regions. The adjacency matrix obtained as a result of this process provides the structural basis for the GNN model to propagate information by reflecting the topological skeleton of the image. This transformation elevates the unit of analysis from individual pixels to regional representations that are semantically richer and computationally more efficient.

After the graph structure is defined, each node is enriched with a comprehensive feature vector encoding the relevant features of that region. Accordingly, five different features have been added to the edge structure. This approach enables the model to learn not only structural relationships but also the intrinsic features of each region. A multi-dimensional feature set is extracted to capture complementary diagnostic clues. The average values in the CIELAB colour space encode subtle differences in intensity and hue, while the normalised geometric centre coordinates encode spatial awareness. Regional texture patterns are analysed at two scales: Local Binary Pattern (LBP) histograms capture micro-patterns, while Haralick features model the spatial relationships of pixel densities. Finally, Hu moments, which are invariant to translation, scaling, and rotation, characterise the shape features of each superpixel. By combining these features, a Node feature matrix is created that contains a rich representation for each node.

In the final stage, the created neighbourhood matrix and node feature matrix form the inputs for the SPX-GNN model. The model, consisting of a series of GNN layers, iteratively updates each node’s representation by collecting information from its neighbouring nodes and combining it with its current representation through the message passing mechanism. This process enables the model to learn both local anomalies and the global relationships between them, allowing it to create richer node embeddings by facilitating information propagation throughout the graph. The updated node representations obtained from the final GNN layer are condensed into a single vector that represents the entire graph via a graph readout layer. This graph-level representation vector is processed through fully connected layers to produce the final diagnostic probability score for the image. Finally, an explainable AI technique based on feature perturbation is applied to interpret the model’s decision mechanism, creating an importance map that highlights the superpixel regions contributing most to the final decision.

## 4. Materials and Methods

### 4.1. Dataset

This study utilised tuberculosis X-ray images commonly used in the literature [33]. The dataset consists of chest X-ray images labelled as either tuberculosis positive or tuberculosis negative (normal), and was created for the training and evaluation of deep learning models. Within the scope of the research, a total of 4200 images from the publicly available part of the dataset were included in the study. Among these images, 700 belong to cases diagnosed with tuberculosis, while 3500 belong to normal chest X-rays. To address the imbalance in class distribution in the dataset and to reliably test the model’s generalizability, the 5-fold stratified cross-validation method was employed for the evaluation process. This method ensures that the class ratios in the training and validation sets remain the same as those in the original dataset for each fold. To enhance the model’s reliability and prevent potential data leakage, a strict pre-processing procedure was implemented. Specifically, feature scaling was not performed on the entire dataset beforehand; instead, it was calculated based solely on the training set in each cross-validation iteration, and the resulting parameters were applied to the validation set. This approach prevented statistical information from the test data from leaking into the training process, ensuring that the performance metrics and confusion matrices obtained were unbiased and reliable.

### 4.2. Image to Graph

The first step in the conversion process is to express the basic representation unit of the image in terms of superpixels rather than pixels. Superpixels are mid-level image units that are perceptually meaningful and generally adhere to object boundaries, created by grouping similar pixels in terms of colour/intensity and spatial location. This approach has two key advantages over pixel-based analysis. First, it increases computational efficiency by reducing the problem’s complexity from millions of pixels to a manageable number of meaningful regions. Second, it enables learning more robust feature representations by focusing the learning process on more stable and statistically more prosperous areas, rather than individual pixels that are more sensitive to noise.

In this work, Simple Linear Iterative Clustering (SLIC) is employed for superpixel segmentation [34]. SLIC partitions an image into a user-specified number, approximately K, of superpixels. The algorithm operates by clustering pixels in a 5-dimensional feature space, which combines the $\left(L^{*},a^{*},b^{*}\right)$ values from the CIELAB colour space and the (x,y) spatial coordinates from the image plane. Using a distance metric that balances the relative importance of colour and spatial proximity, it iteratively assigns pixels to the nearest cluster centres and then updates these centres. Mathematically, given an image I, the SLIC algorithm partitions it into a set of K superpixels:

(1) $$S=\{s_{1},s_{2},\ldots ,s_{K}\},\overset{K}{\underset{k=1}{\cup }}s_{k}=I\;\mathrm{and}\;\forall i\neq j,\;s_{i}\cap s_{j}=\emptyset$$

Each element $s_{k}\in S$ represents a superpixel region. Each of these generated superpixels is then defined as a single node in the constructed graph. Consequently, our node set becomes $V=\{v_{1},v_{2},\ldots ,v_{K}\}$, where there is a one-to-one correspondence between a node $v_{k}$ and a superpixel $s_{k}$.

Once the nodes are defined, the next step is to establish the edges representing their relationships and describe each node with a numerical vector. This process constitutes both the structural topology and the semantic content of the graph. The graph’s structure is determined based on the spatial adjacency relationship between superpixels. If two superpixel regions, $s_{i}$ and $s_{j}$, share at least one common boundary in the original image, an undirected edge is created between the nodes $v_{j}$ that represent them. This adjacency-based approach enables information to flow between locally connected regions, allowing the GNN to model local contexts, such as texture and structure [35]. This topology can be mathematically expressed by an adjacency matrix $A\in {\mathbb{R}}^{KxK}$**:**

(2) $$A_{ij}=\left\{\begin{array}{ll} 1 & \mathrm{if}\;v_{i}\;\mathrm{and}\;v_{j}\;\mathrm{are}\;\mathrm{adjacent}\\ 0 & \mathrm{otherwise} \end{array}\right.$$

Each node $v_{k}\in V$ is endowed with a feature vector $x_{k}\in {\mathbb{R}}^{D}$ that summarises the semantic properties of its corresponding superpixel region $s_{k}$. This feature vector serves as the initial input for the GNN layers. To effectively capture the complex manifestations of tuberculosis, we moved beyond simple colour averages and constructed a comprehensive feature representation by fusing four complementary information types:

1- Colour and Intensity: Instead of the RGB space, we utilised the CIELAB colour space, which is perceptually uniform. We calculated the mean values of the L, A, and B channels for pixels within $s_{k}$ to represent average intensity and colour variations, which are crucial for identifying lesions under varying illumination conditions.
2- Texture Descriptors: Since tuberculosis significantly alters lung tissue texture (e.g., infiltrations or consolidation), we extracted texture features to capture these irregularities. We computed Local Binary Patterns (LBP) histograms (radius = 3, points = 24) to encode micro-texture invariance. Additionally, Haralick features (Contrast, Correlation, Energy, and Homogeneity) were derived from the Grey-Level Co-occurrence Matrix (GLCM) to quantify structural dependencies at the regional level.
3- Shape Invariants: To characterise the geometry of segmented regions independent of their orientation or scale, we calculated the seven invariant Hu Moments for each superpixel mask. This helps the model distinguish specific anatomical shapes regardless of patient positioning.
4- Spatial Location: The normalised centroid coordinates (x,y) were included to allow the GNN to learn position-dependent priors, such as the likelihood of infection in specific lung lobes.

**Figure 2:**
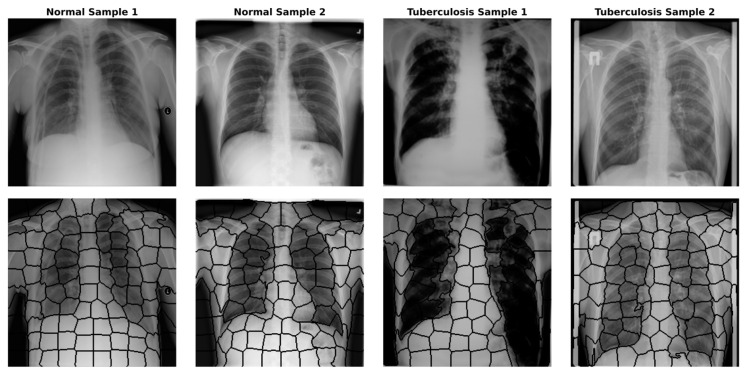
Superpixels obtained by applying the SLIC algorithm to sample images randomly selected from the dataset.

**Figure 3:**
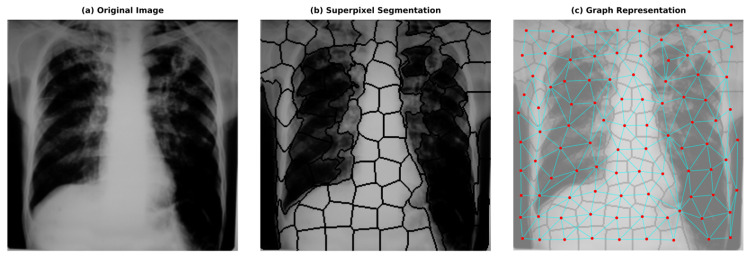
Example showing converting the original image into a graphical representation.

The final node feature vector is obtained by concatenating these feature groups. When this extraction process is repeated for all nodes, a feature matrix $X\in {\mathbb{R}}^{KxD}$ containing the features of all nodes in the graph is formed. Finally, to ensure the stability of the model training and to prevent features with different scales from dominating the learning process, a standardisation operation is applied to the feature matrix derived from all nodes in the training set. At the conclusion of these steps, each input image is transformed into a fully defined graph $G\;=\;\left(V,\;E,\;X\right)$, ready to be processed by the GNN model. Figure 2 shows the superpixels formed by applying the SLIC algorithm to images randomly selected from the dataset. Figure 3 also provides an example image related to converting the original images into graphs.

### 4.3. Graph Neural Network

Once the images are transformed into graph representations that encode structural and semantic information, a deep learning model is required to classify these representations directly. For this purpose, a classifier architecture founded on Graph Convolutional Networks (GCNs) was developed in this study. GCNs are a specific type of GNN that generalises the convolutional operation from regular grid structures, like conventional images, to graph structures with potentially irregular topologies [36,37]. The fundamental objective of the model is to learn a holistic embedding vector that represents the entire graph for classification, which is achieved by enriching the initial features of each node (superpixel) through the propagation of information from its neighbours across the graph structure.

The core building block of the GCN model is the graph convolutional layer. The function of this layer is to compute a new representation, or embedding, for each node by aggregating its current features with those of its direct neighbours. This process is often conceptualised as a message passing scheme. The specific GCN layer implemented in this work is based on the propagation rule proposed by Kipf & Welling, which leverages a symmetrically normalised version of the adjacency matrix to ensure a more stable propagation of node features [38]. This rule is formally expressed as:

(3) $$\;H^{\left(l+1\right)}=\sigma \left({\tilde{D}}^{-\frac{1}{2}}\tilde{A}{\tilde{D}}^{-\frac{1}{2}}H^{\left(l\right)}W^{\left(l\right)}\right)$$

In this formulation, $H^{\left(l\right)}$ is the matrix of node features at layer l, where the process is initialised with the input features $(H^{\left(0\right)}=X).W^{\left(l\right)}$ is the trainable weight matrix for the layer, which transforms the node features into a higher-level latent space. The term ${\tilde{D}}^{-\frac{1}{2}}\tilde{A}{\tilde{D}}^{-\frac{1}{2}}$ represents the normalised adjacency matrix, where self-loops have been added to the original matrix to allow each node to include its features in the update process. This normalisation is crucial as it prevents the feature representations of high-degree nodes from dominating the aggregation and stabilises the numerical computations. Finally, σ denotes a non-linear activation function, such as ReLU. Stacking multiple GCN layers effectively expands the receptive field for each node, enabling a node at the final layer to incorporate information from its multi-hop neighbourhood and allowing the model to learn complex, higher-order structural patterns.

While the GCN layers produce enriched representations at the node level, a single classification decision must be made for the entire graph. This requires a readout or pooling mechanism to aggregate the node-level embeddings into a single vector representing the entire graph. In this work, a masked global average pooling strategy is employed. This method is specifically chosen to handle the fact that graphs generated from different images will have varying nodes [12]. The operation computes a graph embedding, $h_{G}$, by taking the element-wise mean of the feature vectors of all valid nodes from the final GCN layer.

(4) $$\;h_{G}=\frac{1}{K}\sum_{i=1}^{K}h_{i}^{\left(L\right)}$$

Here, $h_{i}^{\left(L\right)}$ is the feature vector of the i−th node at the final layer L and K is the total number of valid nodes in the graph. This resulting vector $h_{G}$ is a fixed-size representation that encapsulates the graph’s structural and semantic properties. This graph embedding is fed into a standard Multi-Layer Perceptron (MLP), consisting of a series of fully connected (Dense) layers. This MLP acts as the final classifier, processing the dense graph representation and producing a probability score between 0 and 1 via a Sigmoid activation function for the binary classification task. The entire model is trained end-to-end using the Binary Cross-Entropy loss function via the backpropagation algorithm.

### 4.4. Perturbation-Based Explainability

Beyond achieving high classification accuracy, a central objective of this framework is to endow the model with transparency, thereby addressing the inherent black box nature of many deep learning architectures. In high-stakes domains such as biomedical analysis, understanding the rationale behind a model’s prediction is as crucial as the prediction itself. To this end, a methodology for explaining the GNN’s decisions is integrated into the pipeline. The approach is based on a perturbation-based explainability technique, which aims to determine the causal contribution of each input component to the final output [39]. Rather than analysing model internals like gradients, this method interrogates the trained model by systematically altering its inputs and observing the resulting changes in its predictions. In the context of our graph-based framework, the fundamental input components are not individual pixels but the semantically meaningful superpixel nodes, allowing for a more interpretable and robust explanation of the model’s behaviour.

The core mechanism of this explainability method involves a form of counterfactual analysis for each node within a given graph. For a specific test image, the model has classified. The process begins by recording the model’s baseline prediction probability for the predicted class using the original, unperturbed graph. Subsequently, the algorithm iterates individually through every valid node in the graph. In each iteration, a temporary, perturbed version of the graph is created by “occluding” the single node under consideration. In this work, occlusion is implemented by masking the node’s influence, which is achieved by setting its entire feature vector to zero. This effectively removes that specific superpixel’s visual and positional information from the model’s view, while keeping the overall graph topology intact. This perturbed graph is passed through the identical, already-trained GCN model, and the new output probability is recorded.

The contribution of each node to the final decision is quantified by calculating an importance score. This score is the difference between the model’s baseline probability and the probability obtained after that node was perturbed [39]. A single, intuitive equation captures this relationship for a given node $v_{i}$:

(5) $$\;\mathrm{Importance}(v_{i})=P(\mathrm{class}|G)-P(\mathrm{class}|G_{\mathrm{occlude}(v_{i})})$$

Here, P(class|G) is the model’s output probability for the original graph, and $P(\mathrm{class}|G_{\mathrm{occlude}(v_{i})})$ is the probability when the graph with node $v_{i}$ occluded is used as input. A high positive importance score indicates that the node provided critical evidence for the predicted class; its removal caused a significant drop in the model’s confidence. Conversely, a score near zero implies the node was largely irrelevant to the decision. A negative score suggests that the node provided counterevidence, and its removal increased the model’s confidence in the final prediction. We generate a comprehensive saliency map by computing this score for every superpixel. This map is then visualised as a heatmap overlaid onto the original image, where the colour intensity of each superpixel corresponds to its calculated importance. This final visualisation provides a direct, human-interpretable illustration of the salient morphological regions that most influenced the GNN’s classification decision.

### 4.5. SPX-GNN Architecture

**Figure 4:**
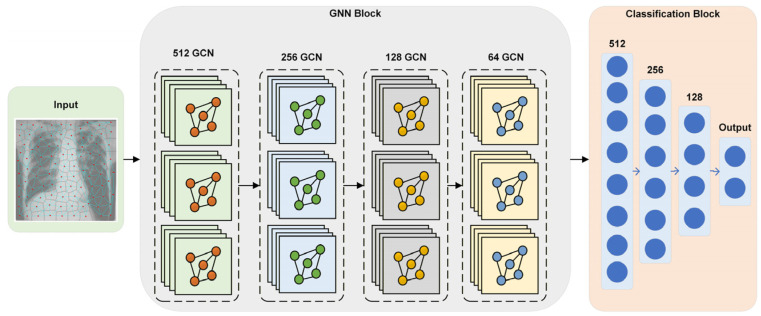
GCN model architecture for SPX-GNN.

The SPX-GNN architecture proposed in this study is a multi-stage deep learning model designed to perform end-to-end learning on the graph representations detailed in the previous section. The fundamental goal of the architecture is to hierarchically learn both the local features of each superpixel node and the global topological structure of the graph formed by these nodes, ultimately producing an accurate and interpretable classification decision for the image. The architecture’s design consists of two main components: a GCN backbone responsible for learning rich feature representations at the node level, and a classification head that performs the final classification by transforming these representations into a holistic graph embedding. The GNN architecture of the SPX-GNN model is given in Figure 4. Additionally, the specific hyperparameters used to configure the model are summarised in Table 1.

The core of the SPX-GNN architecture consists of a four-layer GCN, each comprising 512, 256, 128, and 64 units. This deep architecture is designed to perform hierarchical feature extraction on non-Euclidean graph data. The model’s first GCN layer has a high capacity of 512 units and captures rich and complex local patterns from the initial node features. Subsequent layers gradually reduce the number of units, creating a funnelled structure. This structure lets the model learn more abstract and generalised representations by compressing information in deeper layers. Each GCN layer operates through a message passing mechanism; thus, a node updates its representation by gathering information from its one-step neighbourhood. Stacking four layers allows each node’s final representation to incorporate information from its four-step neighbourhood. The model must model simple relationships between adjacent superpixels, broader tissue architectures, and complex structural dependencies across regions. To reduce the risk of overfitting and increase the model’s generalisation ability, a 10% dropout is applied to the output of each GCN layer. This prevents the model from becoming overly dependent on specific node features or neighbourhood patterns by randomly turning off some neurons during training.

After generating enriched and context-aware feature vectors for each node, the GCN core must convert the node-level information into a single, fixed-dimensional vector representing the entire graph. This process is implemented through a masked global average pooling layer. This layer creates a holistic graph embedding of the graph by averaging all valid node representations from the final GCN layer. This method is ideal for this task because it provides permutation-invariant processing and can naturally handle graphs with varying numbers of nodes across different images. The resulting graph embedding is a dense representation incorporating local superpixel features and their global topological organisation. This embedding is fed into a classification head, a three-layer MLP consisting of 512, 256, and 128 units, respectively. This deep MLP models the complex and non-linear relationships within the graph embedding, ultimately reaching a layer with a single output neuron for the final binary classification task. Similarly to the GCN backbone, a 10% dropout is applied after each fully connected layer to increase the classifier’s robustness against overfitting. This integrated architecture was trained end-to-end using the Adam optimiser and the Binary Cross-Entropy loss function, following the specified training hyperparameters.

## 5. Experimental Results

### 5.1. Evaluation Metrics

The basis of the experimental procedures is the careful preparation of the dataset. To ensure that the model’s training and evaluation processes are independent, the entire image dataset was divided using a stratified k-fold cross-validation. This standard separation is essential to verify that the model does not merely memorise the training data but also learns underlying patterns and can apply them to new samples. The model was trained using the hyperparameter configuration in Table 1 in the previous section. The model weights that showed the best performance throughout the training process were selected based on the highest accuracy value on the test data and recorded for final evaluation. To provide a comprehensive analysis of the SPX-GNN model’s classification performance, performance metrics were used that are capable of capturing the nuances of binary classification tasks. Rather than simply reporting overall accuracy, a series of complementary metrics was calculated to better understand the model’s behaviour. Accuracy provides a snapshot of the model’s overall performance by giving the percentage of correct classifications among all predictions. However, as it can be misleading in scenarios where the class distribution may be imbalanced, it has been supplemented with other metrics. Precision measures how many examples labelled as positive by the model are positive and is a critical indicator in situations where false positive alarms are costly. In contrast, Sensitivity evaluates how many of the examples that are actually positive the model successfully detects and plays a vital role in diagnostic scenarios where it is essential not to miss any positive cases. Complementing Sensitivity, Specificity measures the model’s ability to correctly eliminate negative instances, helping to control the false diagnosis rate.

To achieve a balance between these metrics, the F1-score metric was used. As a harmonic mean of Precision and Sensitivity metrics, the F1-Score provides a more robust assessment of the model’s performance by establishing a balance between these criteria. The Matthews Correlation Coefficient (MCC), recognised as one of the most comprehensive performance measures, was also included in the calculations. The MCC produces a correlation coefficient between *−*1 and +1, considering all four components of the confusion matrix, and is known for being highly robust against class imbalance. Finally, the Zero-One Loss metric directly expresses the model’s error rate and complements the accuracy metric by providing the proportion of misclassified examples. This rich set of metrics, evaluated together on the test set, has enabled the holistic evaluation of not only the overall success of the SPX-GNN model but also its performance for each class, the types of errors, and its potential strengths and weaknesses in practical applications.

### 5.2. SPX-GNN Performance

**Figure 5:**
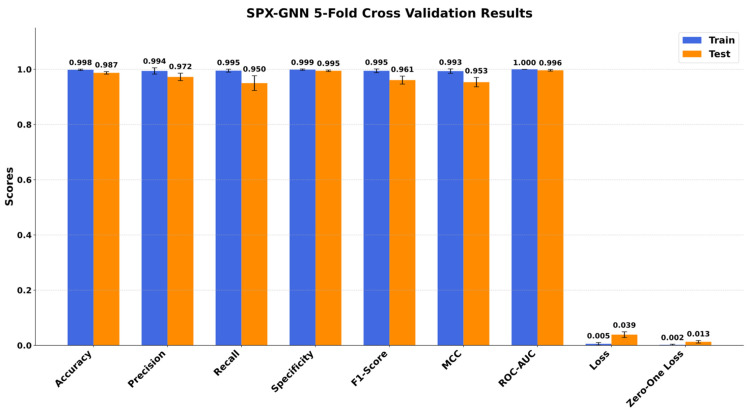
Training and test performance results of the SPX-GNN method.

A comprehensive experimental process was conducted to evaluate the learning capacity, stability, and generalisation ability of the proposed SPX-GNN model. All experimental work was performed on a workstation equipped with an Intel Core i7-11700H CPU @ 4.90 GHz processor, 32 GB RAM, and NVIDIA GeForce GTX 3070 GPU, using the TensorFlow library. The model’s performance analysis was performed using the 5-fold stratified cross-validation method to manage class imbalance in the dataset and ensure the statistical reliability of the results. The quantitative data obtained from the cross-validation process indicates that the performance difference between the model’s training and test sets is relatively low. The fact that the accuracy and loss values calculated for the training and test sets are similar indicates that the model does not overfit solely to the training data, but also achieves high generalisation success on unseen data. Furthermore, the low variation in the metrics obtained across different folds confirms that the proposed architecture and the applied regularisation techniques provide a stable learning process. The results obtained during the model’s training and testing are shown in Figure 5.

The results shown in Figure 5 clearly demonstrate the superior classification performance of the SPX-GNN model as a result of the 5-fold cross-validation process. The model achieved a high average accuracy rate of 98.714% (±0.005) on the test set, proving the stability of its overall classification success. This high accuracy rate is supported by a low average loss value of 0.059 (±0.030). This minimal difference between training and test scores and the low standard deviation values confirm that the model has a strong generalisation capacity and performs a stable learning process without overfitting the data. When class-specific metrics are analysed in depth, the model’s power of discrimination becomes more apparent. In particular, the specificity score of 0.995 (±0.003) obtained on the test set indicates that the model can distinguish tuberculosis-negative (normal) cases with near-perfect accuracy. Similarly, the precision value of 0.972 (±0.014) indicates that the vast majority of cases predicted as positive are indeed positive, proving that the false positive rate is within clinically acceptable limits.

The recall metric, which is of vital importance in biomedical applications, was measured as 0.950 (±0.027); this indicates that the model was able to successfully detect 95% of actual tuberculosis cases. The F1-Score, which expresses the balance between precision and recall, was 0.961 (±0.015), confirming that the model performed well in both classes despite class imbalance. The MCC, one of the strongest indicators of performance robustness, was calculated as 0.953 (±0.017). This value, which is very close to +1, proves that there is an almost perfect correlation between the predicted and actual classes and that the model’s success is independent of chance factors. Finally, the ROC-AUC score of 0.996 (±0.003) shows that the model maintains its discriminatory power even at different threshold values. All these findings lead to the conclusion that the SPX-GNN approach strongly proves the effectiveness of the proposed methodology by demonstrating high accuracy, reliability, and robustness.

**Figure 6:**
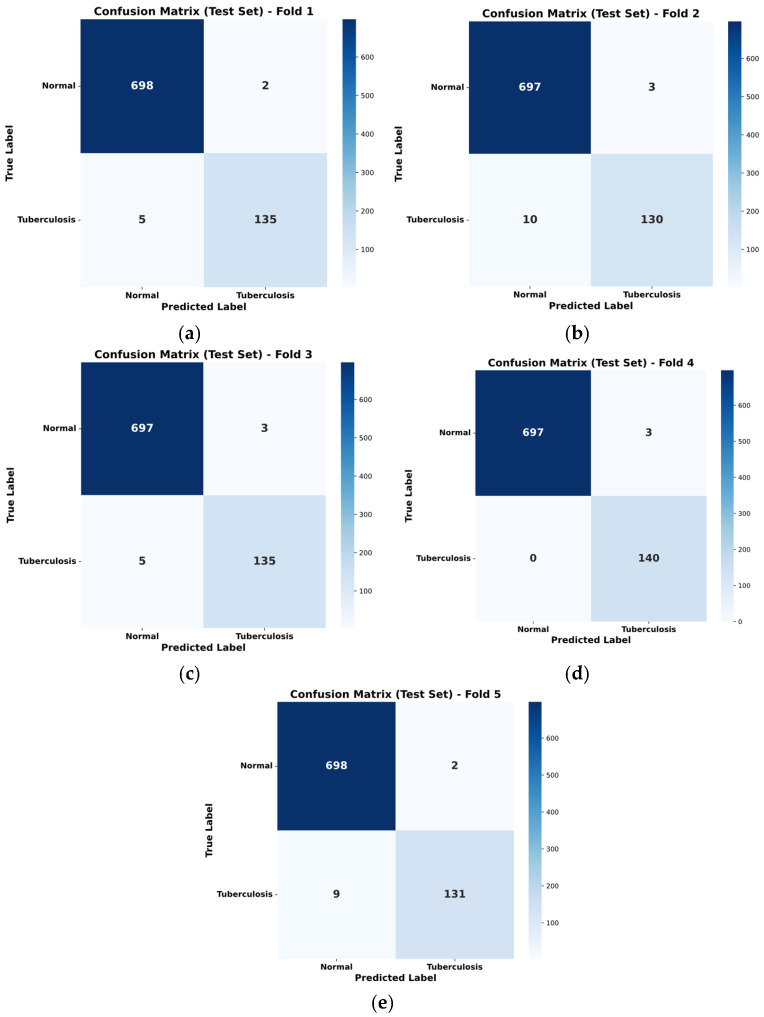
Confusion matrices were obtained for each fold during the k-fold cross-validation experiments. Confusion matrices were obtained for each fold during the k-fold cross-validation experiments, and the matrices corresponding to each fold are presented in (**a**–**e**).

To examine the model’s performance consistency and prediction distribution across classes in greater detail, confusion matrices were analysed separately for each step of the 5-fold cross-validation process. Figure 6 presents the confusion matrices obtained for each fold during the cross-validation experiments. These matrices illustrate that the model exhibits stable behaviour not only in the overall average but also when trained and tested on different subsets of the dataset.

When examining the matrices, it is observed that the model has an extremely high performance rate in classifying examples, particularly in the normal class, and that false positive rates remain minimal. In the tuberculosis class, it was observed that the number of false negatives remained low and stable throughout all folds. The fact that the true positive and true negative rates obtained in each fold are close to each other indicates that the proposed SPX-GNN architecture possesses robust learning capabilities, regardless of the random divisions in the dataset. This analysis confirms that the model’s high accuracy scores are not dependent on a specific data distribution or chance, but rather stem from the method’s generalizable characteristics.

### 5.3. Ablation Study

**Figure 7:**
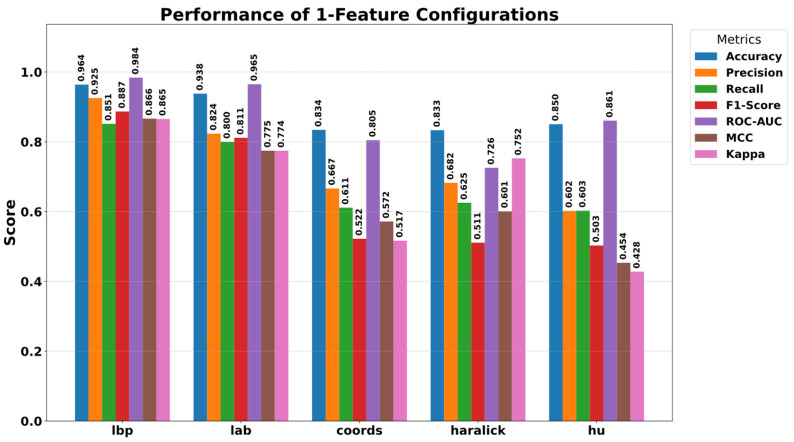
Performance results obtained by using singular features.

A comprehensive ablation study was conducted to reveal the contribution and necessity of the components that constitute the high classification success of the proposed SPX-GNN model. In this study, the effect of each feature group integrated into the model on the model’s decision mechanism was systematically analysed, from individual use to integrated structure. In the first stage, the performance provided by the feature groups, independent of the model, was examined, and the results obtained are presented in Figure 7. When evaluating the individual feature performances, it is seen that the LBP (96.38% accuracy) and LAB (93.80% accuracy) features provide the strongest baseline performance. This suggests that local tissue patterns and colour information are the most discriminative features in tuberculosis detection. In contrast, Coordinate (83.42%), Haralick (83.33%), and Hu moments (85.04%) showed more limited success when used alone. The results in Figure 7 indicate that structural and positional features are not sufficient on their own, but become meaningful when supported by tissue and colour information.

**Figure 8:**
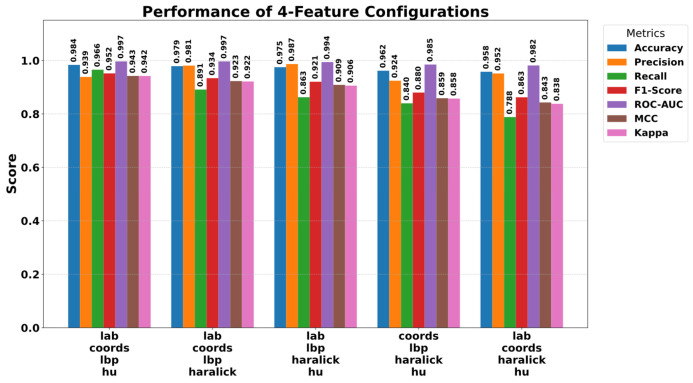
Performance results obtained using the four features.

To more clearly observe the complementary nature of the features and their necessity for the model, four combinations were created by removing one feature group at a time from the complete model. The findings are visualised in Figure 8. This analysis strikingly reveals how performance is affected in the absence of specific components. Specifically, in the configuration where LBP features were removed from the model (lab, coords, Haralick, and Hu), the accuracy rate dropped to 95.80%. This decline demonstrates that LBP forms an indispensable foundation for the model’s overall decision-making mechanism. A more specific effect was observed in the scenario where Hu moments were removed (lab, coords, LBP, and Haralick). As shown in Figure 8, although the overall accuracy remained at 97.90% in this case, the Recall value experienced a sharp decline from 0.9987 to 0.8914. This critical finding demonstrates that Hu moments play a vital role in defining the morphological structure of tuberculosis-positive cases and preventing missed cases.

**Figure 9:**
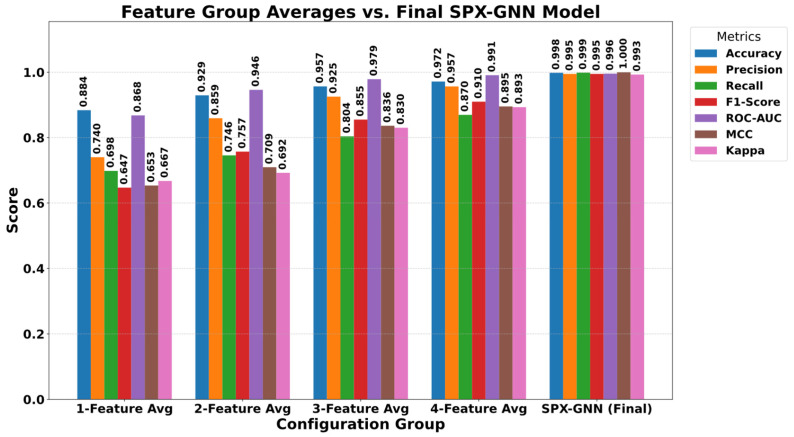
Performance comparison using the SPX-GNN model based on the average values of ablation variants.

In the final stage, the overall performance of the SPX-GNN model, which integrates all feature groups, was evaluated, and the comparative results with other variants are presented in Figure 9. This final model, which combines colour, shape, texture, and position information in a hybrid structure, demonstrated the best performance among all evaluated configurations. The SPX-GNN model achieved success beyond individual or reduced combinations, with an accuracy of 0.9982, precision of 0.995, sensitivity of 0.998, and an F1-score of 0.995. Furthermore, the obtained ROC-AUC value of 1.000 and MCC value of 0.993 confirm that the model’s class separation ability and generalizability are at the highest level. In conclusion, this study, supported by Figure 9, provides scientific evidence for the necessity of strategically combining multifaceted and complementary feature sets, rather than relying on a single feature group, for the detection of complex pathologies such as tuberculosis.

### 5.4. Explainability Results of SPX-GNN

**Figure 10:**
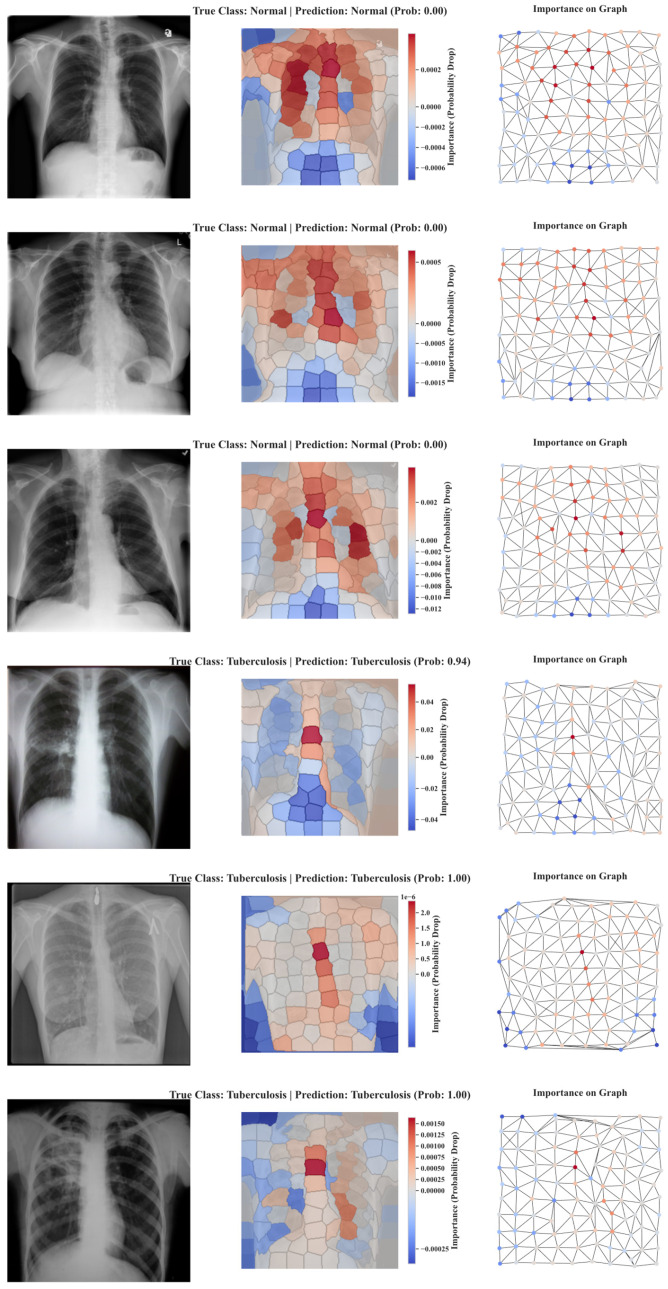
Visualisation of the SPX-GNN model using xAI. It shows the contribution of the atoms and nodes of the input images to the prediction class.

The superior quantitative performance metrics presented in the previous sections demonstrate the success of the SPX-GNN model. However, understanding how and why the model arrives at these decisions is equally important, especially in fields where trust and interpretability are critical, such as biomedical image analysis. To this end, a perturbation-based explainability technique was applied to examples in the validation set to shed light on the model’s decision-making mechanism and eliminate its black box nature. Technically, this process begins by recording the model’s initial prediction probability for a test image. Then, a perturbation operation is applied sequentially to each superpixel node forming the graph. In this study, perturbation is an occlusion operation performed by temporarily replacing the feature vector of the relevant node with zeros. This operation effectively hides the semantic content of that superpixel, such as colour and position, from the model while preserving the graph’s overall topological structure, i.e., its edge connections. Each time a node is occluded, this modified graph is passed through the same trained model again, and the new prediction probability is recorded. The final importance score of a node is calculated as the difference between the initial probability and the probability after that node is occluded. Therefore, if removing a node causes a significant drop in probability, it is concluded that the node provides critical positive evidence for the model’s decision. Figure 10 shows the importance maps generated for correctly classified examples from both classes.

The qualitative analysis of these maps provides in-depth insights into the decision strategy of the SPX-GNN model. When examining samples correctly classified as tuberculosis, it is observed that the model consistently focuses its attention on specific regions expected to exhibit pathological abnormalities. Super-pixels with high positive scores, shown in red on the saliency maps, are concentrated in areas with dense or irregular tissue patterns that typically differ from healthy tissue regions. This indicates that the presence of these regions significantly increases the likelihood of the model favouring the tuberculosis class. The model’s focus on structural abnormalities that correspond to the distinctive morphological features a radiologist would look for powerfully demonstrates that the GNN architecture learns not only superficial tissues but also inter-regional contextual relationships.

On the other hand, importance maps generated for examples commonly classified as correct reveal a different, yet equally logical, decision strategy. In these examples, high importance scores are generally concentrated in tissue regions that are homogeneous and structurally consistent, showing no pathological signs, or sometimes in background areas that lack meaningful structure. This situation reveals that the model bases its normal decision not on the presence of an abnormality but on the absence of pathological indicators. The model increases its confidence in the normal class by confirming that a large portion of the image is consistent with the expected healthy tissue pattern. Another interesting point in this analysis is the blue-coloured negative importance scores observed in some maps. A superpixel with a negative score represents an area where the presence of the object slightly reduces the model’s confidence in the correct class. These regions may contain confounding features such as image artefacts, lighting irregularities, or ambiguous tissue patterns. The model’s assignment of negative importance to these regions demonstrates that it actively detects these misleading signals and gives them less weight in the decision process, providing further evidence of the robustness and sophistication of the architecture.

In conclusion, this explainability analysis conducted for SPX-GNN demonstrates that the model possesses a logical and consistent internal reasoning mechanism beyond being merely a high-accuracy classifier. Saliency maps indicate that the model relies on concrete visual evidence that experts can verify and interpret. This transparency enhances trust in the model and significantly increases its value in practical clinical and research applications, potentially enabling the discovery of new biomarkers or areas of interest for future research.

### 5.5. SOTA Comparison

To determine the effectiveness of the developed SPX-GNN model in tuberculosis classification and its place in the literature, a comparative analysis with state-of-the-art (SOTA) studies was conducted. This comparison is summarised in Table 2. The standard criterion for the studies included in the evaluation was that they targeted the tuberculosis diagnosis problem. Numerous successful results have been obtained in the literature using different datasets and deep learning architectures. Therefore, to evaluate the performance of the proposed model from a broader perspective, this table includes fundamental papers that focus directly on the tuberculosis classification task and report high-performance results, rather than a limited group of studies using the same dataset.

As shown in Table 2, a wide range of deep learning models, including DenseNet [33,48], ViT [14], NFNets [41], VGG-variants [44,46,47] and various CNN [40] and ANN [47] architectures have been proposed for tuberculosis detection. These studies have achieved high state-of-the-art (SOTA) results with their accuracy values. Compared to these strong results, our proposed SPX-GNN model has set a new threshold in classification performance, surpassing all reference studies listed in the table with a 99.82% accuracy rate. The most striking advantage of our model is its precision value of 99.40%. This metric indicates that all cases classified as positive by the model are correct, meaning the false positive rate is extremely low, which is critically important in clinical applications for preventing unnecessary treatments or biopsies. Furthermore, the SPX-GNN model achieved a better precision-sensitivity balance than other strong models with a 99.45% F1-Score. The model’s 100.00% ROC-AUC value is significantly higher than values from other studies, proving that the model has superior discrimination ability even at different threshold values. This comprehensive comparative analysis powerfully demonstrates that the SPX-GNN approach offers higher accuracy, excellent precision, and exceptional overall performance compared to current SOTA methods in the task of tuberculosis classification.

## 6. Conclusions

The success of deep learning methods is constrained by fundamental architectural assumptions that treat data as a Euclidean grid and operate on local regions. This structural limitation prevents the modelling of the holistic context between spatially dispersed findings, which are frequently encountered in medical images and are diagnostically critical. This work addresses this fundamental issue by enabling image analysis by transforming Euclidean data into a non-Euclidean space. The proposed SPX-GNN framework converts images into a graph of semantic superpixel nodes. It processes this representation with a GNN, enabling the learning of these long-range relational patterns. The proposed SPX-GNN method represents each semantic region, the superpixels, with a multi-modal feature signature encoding complementary diagnostic clues such as colour, texture, shape, and spatial location. This rich node representation has enabled the model to learn local anomalies and the global relationships between these findings through the GNN’s message passing mechanism. This approach allows the model to base its decision-making process on a holistic pattern formed by scattered and multifocal findings, thereby achieving a level of reasoning that traditional ESAs fall short of. The proposed SPX-GNN method represented each semantic region with a multidimensional feature signature encoding complementary diagnostic clues such as colour, texture, shape, and spatial location. The effectiveness of this comprehensive feature set was systematically validated through ablation studies. These experiments demonstrate that each feature category contributes meaningfully and complementarily to the model’s overall performance, with the highest success achieved using a holistic model that combines all features. These findings emphasise the critical importance of rich feature representation for capturing the multidimensional nature of medical abnormalities.

Experimental results strongly validate the effectiveness and superiority of the proposed SPX-GNN model. SPX-GNN achieved an accuracy of 0.998, an F1-score of 0.994, and a nearly perfect ROC-AUC value of 1.000 in classifying tuberculosis lung radiographs. In particular, the precision score of 0.994 indicates that every case identified as positive by the model is unquestionably correct, demonstrating its success in minimising the false positive rate. These metrics demonstrate that SPX-GNN provides high diagnostic accuracy and highly reliable, robust performance. In addition to the experimental success of the SPX-GNN method, one of the key contributions of this work is to increase model interpretability. SPX-GNN incorporates an xAI module that illuminates the decision mechanism at a semantic level. This layer eliminates the black box nature of the model by generating saliency maps that highlight the superpixel regions contributing most to the final diagnosis. This capability is vital not only for model validation and error analysis but also for building trust in clinical decision support systems and potentially enabling the discovery of new diagnostic biomarkers. In conclusion, this study successfully demonstrates the power of combining GNNs and xAI techniques for biomedical image analysis. SPX-GNN offers a potential roadmap for specific tasks, such as tuberculosis diagnosis, and a broader range of problems where structural and topological information is critical, including digital pathology and neuroimaging. Future work could extend this framework to apply it to different diseases and imaging modalities, integrate richer node features, and explore more advanced graph learning architectures. Based on the current findings, the flexible and modular structure of the proposed SPX-GNN methodology demonstrates that the method is not limited to chest radiographs but can be easily adapted to different medical imaging modalities. Particularly in areas such as histopathological image analysis, brain MRI network classification, or retinal imaging, where inter-pixel connectivity and topological structures are critical for diagnosis, the proposed graph-based approach has the potential to offer a powerful alternative to traditional CNN-based methods. Furthermore, since superpixel-based graph representation enables working with images of different resolutions and scales, the proposed architecture has the capacity to be effectively applied in large-scale clinical studies using multi-centre and heterogeneous datasets.

## Figures and Tables

**Table 1:** Specific hyperparameters used in configuring the SPX-GNN method.

	Parameter	Value	Description
Image to Graph	Super-pixel Number	100	The number of superpixels (nodes) targeted by the SLIC algorithm for each image.
	Super-pixel Compactness	25	Adjusts the balance between colour and spatial proximity. Higher values produce more square superpixels.
	Gauss Filter Sigma	0.9	Standard deviation of the Gaussian smoothing applied to the pre-SLIC image.
GNN Model Architecture	GCN Layer Size	512, 256, 128, 64	Kernel size of four GCN layers
	Dense Layer	512, 256, 128	Number of dense layers following the pooling layer
	Dropout Rate	10%	The dropout rate is applied after each GCN and dense layer to prevent overfitting.
Training and Optimisation	Learning Rate	0.001	The value that determines the step size of the optimiser.
	Batch Size	32	The number of graphs used in each training iteration.
	Maximum Epoch	100	The maximum number of iterations the model performs on all training data.
	Optimisation Algorithm	Adam	An adaptive momentum-based optimisation algorithm for gradient descent.
	Loss Function	Binary Cross-Entropy	Standard loss function for binary classification problems.

**Table 2:** Performance comparison of SPX-GNN’s performance metrics with state-of-the-art models. (The best-performing results are highlighted in bold).

Reference	Algorithm	Accuracy (%)	Precision (%)	Sensitivity (%)	F1- Score (%)	ROC-AUC (%)
[14]	ViT-GradCAM	97.00	99.00	99.00	98.00	-
[33]	DenseNet	98.60	98.56	98.56	-	-
[40]	CNN	96.71	-	-	-	-
[41]	NFNets	95.91	91.67	91.78		98.32
[42]	Transfer Learning	92.6	-	-	-	-
[41]	NF net model	96.91	91.81	98.42	-	99.38
[43]	ResNet	88.24	88.42	88.00	-	93.00
[44]	VGG-16	89.77		90.91	-	-
[45]	VGG-19		81.50	96.20	-	92.00
[46]	VGG19 + CNN	96.48	93.75	97.56	95.62	99.82
[47]	ANN	98.45	98.01	96.12	95.88	-
[48]	DenseNet	98.80	94.28	98.50	96.35	-
**SPX-GNN (Proposed Method)**	**GNN**	**99.82 ± 0.002**	**99.40 ± 0.011**	**99.50 ± 0.005**	**99.45 ± 0.006**	**100.00 ± 0.000**

## Data Availability

The dataset utilised in this study is publicly available and was obtained from [33]. The proposed models, model source code, model application code, fine-tuning, and the scripts required to reproduce the result are available in GitHub public repositories (https://github.com/pala2515/SPX-GNN).

## Abbreviations

The following abbreviations are used in this manuscript:

SPX-GNN	Superpixel Explainable Graph Neural Network
xAI	Explainable Artificial Intelligence
GNN	Graph Neural Network
CNN	Convolutional Neural Network
ViT	Vision Transformer
LBP	Local Binary Pattern
SLIC	Simple Linear Iterative Clustering
GCN	Graph Convolutional Network
MLP	Multi-Layer Perceptron
ANN	Artificial Neural Network
ROC-AUC	Receiver Operating Characteristic–Area Under the Curve
